# Experiences of pressure to conform in postgraduate medical education

**DOI:** 10.1186/s12909-017-1108-8

**Published:** 2018-01-03

**Authors:** Jan Grendar, Tanya Beran, Elizabeth Oddone-Paolucci

**Affiliations:** 1 Department of Postgraduate Medical Education, Cumming School of Medicine, University of Calgary, Calgary, Canada; 2 Department of Community Health Sciences, Cumming School of Medicine, University of Calgary, 3280 Hospital Drive, NW, Calgary, AB T2N 4Z6 Canada; 3 Departments of Community Health Sciences and Surgery, Cumming School of Medicine, University of Calgary, Calgary, Canada

**Keywords:** Group conformity, Multidisciplinary, Patient safety, Peer pressure, Postgraduate education, Teamwork

## Abstract

**Background:**

Perception of pressure to conform prevents learners from actively participating in educational encounters. We expected that residents would report experiencing different amounts of pressure to conform in a variety of educational settings.

**Methods:**

A total of 166 residents completed questionnaires about the frequency of conformity pressure they experience across 14 teaching and clinical settings. We examined many individual characteristics such as their age, sex, international student status, level of education, and tolerance of ambiguity; and situational characteristics such as residency program, type of learning session, status of group members, and type of rotation to determine when conformity pressure is most likely to occur.

**Results:**

The majority of participants (89.8%) reported pressure to conform at least sometimes in at least one educational or clinical setting. Residents reported higher rates of conformity during informal, rather than formal, teaching sessions, *p* < .001. Also, pressure was greater when residents interacted with higher status group members, but not with the same or lower level status members, *p* < .001. Effect sizes were in the moderate range.

**Conclusions:**

The findings suggest that most residents do report feeling pressure to conform in their residency settings. This result is consistent with observations of medical students, nursing students, and clerks conforming in response to inaccurate information within experimental studies. Perception of pressure is associated with the setting rather than the trainee personal characteristics.

**Electronic supplementary material:**

The online version of this article (10.1186/s12909-017-1108-8) contains supplementary material, which is available to authorized users.

## Background

In an era of sub-specialization and rapid growth of evidence based medicine, it is becoming increasingly difficult for just one physician to assure the most up-to-date patient care. Multidisciplinary teams have been created in response to this change. The importance of this shift in our health care system is evident in competency frameworks, such as the Royal College CanMEDS roles [1]. This framework describes core competencies of Canadian specialist physicians, one of which is Collaborator whereby physicians work effectively within a healthcare team to achieve optimal patient care [2]. Potential pitfalls associated with this trend towards multidisciplinary healthcare teams include ineffective communication and decision-making. More specifically, team members may feel the pressure to conform to others, creating reluctance to propose alternative ideas. This type of pressure may be particularly acute for residents as they encounter various learning situations. Examining this possibility is the aim of this research.

Residency education typically utilizes formal and informal sessions that address specialized areas. In addition to the formal sessions such as scheduled educational sessions, there are many informal teaching opportunities that occur during daily clinical activities – bedside teaching during rounds, work in clinics and in the operating room. Both formal and informal “work-based learning” [3] play an important role in daily acquisition of knowledge and skills. To promote knowledge retention, active discussion of trainees in either type of teaching session is key [4]. Certainly, this discussion can give rise to difference of opinion in clinical medicine. In this clinical environment where trainees strive to show mastery and deference towards their educators, their expression of disagreement in these discussions may be inhibited. Clearly, it would be more comfortable for residents to agree with, rather than question, information that seems inaccurate. Known as conformity, this construct has been thoroughly studied in social psychology; it has received less attention in medical education. This study examines residents’ perceptions of the pressure to conform to inaccurate information, and factors associated with this pressure. Conformity is critical to study, as it may directly interfere with residents’ learning and retention of accurate information.

### Conformity

Conformity refers to matching one’s behavior or opinion to the group consensus [5, 6]. Indeed, people regularly compare themselves with others to determine the appropriateness of their behavior, and this comparison creates pressure to succumb to others’ behaviors [7]. Although the positive influence of conformity may increase adherence to good medical practice, it may also pressure trainees to follow inaccurate information. Research emerging in medical education reveals this to be the case. In the first experimental study of conformity among clerks, Beran and colleagues observed that the majority of clerks inserted a needle in the wrong location during a simulation of knee arthrocentesis upon seeing needle holes in the same wrong location [8]. Similar results were found among medical and nursing students whereby the majority of both groups reported inaccurate vital signs upon hearing those same values reported by confederates in an experimental study during simulation [9]. Physicians too have been identified as sometimes “going with the flow” [10]. These and other studies provide compelling evidence that conformity is an underlying pressure in medicine and medical training [11–14].

### Factors associated with conformity

There are several factors that may increase the likelihood of residents feeling pressure to conform. These can be classified as individual and environmental characteristics. The former may include age, whereby younger participants are shown to conform more often than older participants [15]. Perhaps with increasing age, people are less concerned about being judged on their own opinion [16]. Multiple systematic reviews and meta-analyses evaluated the relationship between sex and conformity [17–19]. Overall, higher rates of conformity are found among female compared to male participants; however, the size of the effect is small, and some studies do not find this difference [17, 20]. Level of education, while related to age, may also be relevant. It is plausible that students with less knowledge and experience are more likely to conform than more senior students, perhaps due to self-doubt and limited mastery [12]. Also, residents who are international medical graduates (IMGs) may feel greater pressure to belong, and, thus, conform more than local graduates [14]. Finally, an individual’s ability to tolerate ambiguity may be relevant. That is, people may feel greater pressure to conform when they feel they need definitive solutions and more certainty compared to those who experience less conformity pressure [17]. In regards to environmental factors, the size of the group seems to influence pressure to conform. When an individual hears dissenting information from three or more, he or she is more likely to conform than when in smaller groups [21]. Also, status within the group is relevant whereby less senior people may conform to more senior colleagues with the former feeling more confident in their experience than the latter [13, 22, 23].

### Purpose of the study

Although research has provided considerable evidence that conformity occurs, there is a gap in understanding the extent to which residents experience it and why it occurs. This study is an exploratory examination of possible individual and situational characteristics that are associated with the perceived pressure to conform. The aims of this study were to 1) describe postgraduate trainees’ perceptions of the pressure to conform, 2) identify individual and situational factors that are associated with these perceptions and to 3) understand the perceived impact of conformity on residents’ acquisition of knowledge and skills.

We decided to measure perception of pressure to conform rather than conforming, per se, as conforming in an individualistic society within a hierarchical profession is generally considered socially undesirable [24]. Also, perceptions provide insights into people’s experiences and level of awareness of conformity. To reduce the problem of under-reporting, we asked participants to indicate when and where they felt the pressure to conform. In addition, we examined individual characteristics related to conformity pressure (age, sex, year of residency, international student status, and tolerance of ambiguity), as well as situational factors (group size, seniority within the group). Also, specialty in which residents are training may be relevant whereby, based on our personal observations, we expected residents in surgical compared to non-surgical programs to feel greater pressure to conform; and residents in their home program rotations to feel more confident than residents in an off-service rotation. Given the foregoing empirical evidence that conformity to inaccurate information does occur in experimental conditions in medical training, it is important to determine residents’ perceptions of the pressure to conform, particularly, as they near independent practice. In particular, medical care must be informed by sound judgement to prevent medical errors – the third most common cause of death in the United States [25].

## Methods

This is a cross-sectional study using a questionnaire designed to describe the extent of self-reported perception of conformity pressure among residents. Participants were enrolled in accredited postgraduate residency training programs at a Canadian University. Administrators of all 55 accredited Postgraduate Medical Programs were contacted. Seventeen program directors responded and during a total of 18 weekly academic half day sessions of those respective residency programs a time slot was scheduled to introduce the research project and to distribute questionnaires. During these sessions, 176 residents were present (out of 750 residents enrolled in all 55 accredited programs). A total of 166 residents provided completed questionnaires. Thus, the response rate was 94.3%. This sample represents 22.1% (166/750) of the total population of residents enrolled in the accredited postgraduate programs at the university.

### Participants

A sample size calculation was performed a priori using the G*Power software [26], version 3.1.9.2. A total of 172 participants was determined to be sufficient to achieve power of 90% at a significance level of.05 for a two-tailed test to detect a moderate effect size of *d* = 0.5 for comparing groups. Due to incomplete questionnaires, a final sample of 166 was obtained.

### Questionnaires

The questionnaire designed for this study is found in Additional file 1. The first section consists of 21 questions describing participants’ age, sex, level of training, residency program, IMG status, and tolerance for ambiguity. IMG status included Canadian IMGs (CIMG) - Canadian citizens who attended medical schools abroad (most commonly in the Caribbean, Australia or Europe). By definition, those that graduated from Canadian medical schools were not in the IMG/CIMG category regardless of their nationality. Tolerance was assessed using the Tolerance for Ambiguity scale developed by Herman [27]. It is a 12-statement questionnaire with 5-point Likert scale answers where 1 = “strongly disagree”, 2 = “disagree”, 3 = “neither agree nor disagree”, 4 = “agree”, or 5 = “strongly agree”. Higher scores indicate higher tolerance for ambiguity. The reported reliability of item scores is 0.76, according to Cronbach’s alpha [27], and in our study it was calculated to be 0.70. This measure is applicable across cultural groups, age, and occupations [28].

The second section of the questionnaire starts with a description of conformity, followed by a resident’s estimate of clinical decisions made under such pressure, followed by 14 scenarios of various formal and informal training experiences in postgraduate medical education. These scenarios include clinical rounds, discussions with peers, operating room encounters and academic half days. To assess the role of hierarchy during teaching encounters, this second section evaluates pressure to conform when participants interacted with others at various levels of expertise (i.e., staff, fellows, senior and junior residents, medical students, observers, nurses, other health care professionals), as well as patients and their families. Participants were asked to assess each scenario twice – once as it pertained to their on-service, and then once as the scenarios related to mandatory off service rotations (for example a surgical resident during their internal medicine or anesthesia rotations). Respondents were asked to evaluate the 14 settings according to how often they felt pressure to conform in each one, using a Likert scale: “almost never” = 1, “rarely” = 2, “sometimes” = 3, “often” = 4, “almost always” = 5. High scores indicate high rates of pressure to conform. The next section lists questions about the specific experiences residents have had. Due to limited responses, these data were not analyzed. The final section consists of two questions about the severity and two questions about the impact of conformity pressure, also rated on Likert scales. Content validity for the items was obtained through input and editing from residency directors and researchers in medical education. The questionnaire was then pilot tested with five residents to ensure accurate interpretation of items.

During the regularly scheduled half day, a 30-min block of time was secured for implementation of the questionnaire. This period consisted of a 10 min introduction of the study and questionnaire followed by a 10 min self-administration of a paper copy of the questionnaire. Participation in the study was voluntary. All of the questionnaires were collected immediately after completion.

### Analyses

All statistical analyses were performed using IBM SPSS® V.22 software. Descriptive analyses were used to summarize resident and program characteristics, as well as reports of conformity pressure. To identify factors related to conformity, Pearson’s Product Moment correlations, t-tests, and repeated measures were calculated for continuous variables. The latter analysis was used for the question about interactions with people of varying status as presumably they would have interacted with all the status groups. Bonferroni corrections were performed to control for the multiple comparisons.

Several variables were recoded into a smaller number of categories to summarize the data. That is, training level was dichotomized into junior resident (PGY level 1–3) and senior resident (PGY 4+) groups. Residency programs were dichotomized into surgical and non-surgical programs. Settings were divided into formal and informal categories, with only academic half days coded as formal. Informal discussions with peers, clinical rounds and teaching in the operating room were regarded as informal sessions. To describe the possible effect of hierarchy, five categories were created - lower rank (medical students, lower PGY residents), same rank (same PGY residents and psychologically near residents described as “good friends”) and higher rank medical professionals (higher PGY residents and staff physicians), nurses and other health care professionals, and non-medical participants (patients and their families).

## Results

### Participant characteristics

The mean age of the respondents was 31 years (25–51 years) and most were female (see Table 1). The majority of participants were junior residents, with over a third of the participants at the program year (PGY) 1 level of training. The majority of respondents belonged to residency programs of over 20 enrolled. Almost 20% of the participants attended medical school outside of Canada.

**Table 1:** Resident characteristics (*N* = 166)

Characteristics	*N* (%)
Female sex	86 (55.5)
Missing	11 (6.6)
PGY 1	58 (35.9)
PGY 2	25 (15.4)
PGY 3	24 (14.8)
PGY 4	23 (14.2)
PGY 5	19 (11.7)
PGY 6, 7, 8	13 (8.0)
Missing	4 (2.4)
IMG	25 (15.1)
CIMG	8 (4.8)
Small size program (<6 residents)	10 (6.0)
Medium size program (6–20 residents)	54 (32.5)
Large size program (>20 residents)	102 (61.5)
Non-surgical programs	99 (59.64)

PGY = postgraduate year of training; IMG = International Medical Graduate; CIMG = Canadian International Medical Graduate

### Rate of conformity

Of the 166 participants, a total of 149 (89.8%) identified the pressure to conform occurring at least sometimes or more often, i.e., ratings of three or more on the five-point scale, in at least one postgraduate setting. Moreover, in their initial estimate they reported that the mean rate at which they experienced this pressure was in 28.65% of the residency situations where decisions are made. The highest frequency of reported pressure to conform occurred during the on-service operating room (mean frequency of 2.88, *SD* = 1.19), several off-service situations such as with a preceptor (mean frequency of 2.87, *SD* = 0.96), in the operating room (mean frequency of 2.79, *SD* = 1.24), and in clinical encounters (mean frequency of 2.78, *SD* = 1.00). On-service clinical rounds were associated with a mean score of 2.58, *SD* = 0.92, signifying the experience of conformity as occurring “sometimes”. The ratings of mean conformity pressure for the remaining situations were 2.12, *SD* = 0.96 to 2.40, *SD* = 0.93, which means conformity pressure was rarely experienced. The mean rating for the severity of conformity was 2.31 (*SD* = 0.83) and the effect on perceived medical errors was 1.75 (*SD* = 0.71). In terms of the impact on knowledge, the mean was 2.98 (*SD* = 0.70) and the impact on skills developed was a mean of 3.04 (*SD* = 0.72).

### Factors related to conformity

Individual and situational factors are shown in Table 2. There were no significant differences in rates of perception of conformity pressure for any resident characteristics, including age (*r* = −0.06, *p* = .43), year of residency (*F*(7154 = 0.85, *p* = .55), sex (*F*(1153) = 1.90, *p* = .17), international status (*F*(1164) = 2.55, *p* = .10), or tolerance of ambiguity (*r* = −0.08, *p* = .30). In regards to situational characteristics, conformity pressure perception did not differ significantly for surgical programs, size of program, or on/off service rotations. However, two characteristics were significant. That is, students perceived greater pressure to conform during informal, compared to formal, sessions (*p* = 0.000). Also, hierarchy of group members was significant (*p* = 0.000), whereby residents reported experiencing more pressure when with higher status residents, same level residents, patients, and nurses compared to lower status residents. In summary, the pressure to conform was likely to be experienced during informal educational encounters, and when with any group other than lower year residents.

**Table 2:** Mean pressure to conform based on environmental characteristics

Groups	Mean (SD)	*p*
Surgical and non-surgical programs *t* (164) = 0.43		= .67
Surgical	2.27 (0.63)	
Non-surgical	2.31 (0.55)	
Size of program *F*(2163) = 0.37		= .69
<6 residents	2.33 (0.41)	
6–20 residents	2.24 (0.58)	
>20 residents	2.32 (0.60)	
Type of educational encounter *t* (50) = −5.50, d = 0.59		= .000
Formal	2.17 (0.79)	
Informal	2.65 (0.83)	
Participants/hierarchy *F*(3.32, 255.86) = 21.30, *partial eta squared = 0.22		= .000
Clerks or lower year residents	1.77 (0.73)	
Same year or psychologically near residents	2.26 (0.73)	
Patients and their families	2.24 (0.85)	
Nurses or other health care professionals	2.31 (0.88)	
Higher year residents or staff	2.49 (0.66)	
Rotation type *t*(21) = 1.97		= .06
Home service	2.24 (0.58)	
Off-service	2.21 (0.58)	

*Note Mauchly’s Test of Sphericity was significant thus Greenhouse-Geisser is reported

## Discussion

This study is the first to describe residents’ perspectives about conformity, with over three-quarters of the residents surveyed reporting this pressure, occurring in over a quarter of the clinical decisions made. This rate is similar to experimental studies of medical students, nursing students, and clerks performing clinical skills poorly in accordance with others’ demonstration of poor skill [8, 9]. Moreover, students are also likely to use these incorrect behaviors to inform diagnoses [29]. Combined with decades of research in social psychology [11], the present results add further evidence of the universality of the conformity phenomenon both within and outside of medical education. Many reasons have been suggested as to why people “go along with” information they consider incorrect. These include the desire to be seen as knowledgeable and competent during evaluation, a need to gain group membership, and a quick response to time pressure [12, 30]. Anecdotally, residents are known to strive at developing mastery and establishing their role within a time constrained medical system.

Interestingly, the questionnaire we used did not identify any individual resident characteristics related to the perceived pressure to conform. This finding suggests that we cannot readily determine which residents are most at risk of feeling this pressure. Perhaps most residents perceive barriers and reluctance to communicate their opinions during educational encounters in their respective programs. There are several potential explanations. Perhaps all residents experience some degree of doubt as they are in the process of developing competence in medicine. This doubt may make them sensitive to pressure to follow what others in their educational and clinical environments say and do. It is also possible, that since all residents experienced the same selection process to admission into medical school, candidates with similar qualities may have been selected [31]. Also, having been exposed to similar training during medical school, they may have adopted comparable perceptions of pressure [32].

Rather than individual characteristics, conformity pressure was found to be related to situational ones. That is, it most often occurred during on-service operating room, followed by off-service preceptor, off-service operating room, and clinical off-service encounters. The off- service encounters may induce the most pressure to conform because of a pronounced power difference during these encounters. Off-service rotations usually occur during junior level training years. In addition, an unfamiliar environment, colleagues and disease pathophysiology create predictable power differences when discussing details of diagnostic and therapeutic plans. The surprising result is the overall highest perceived pressure to conform during on-service encounters in the operating room. Certainly, there is a high power differential with high demands for acuity and accountability. It is unclear why the off-service operating encounters were not also rated as highly.

Despite the general belief that informal teaching sessions offer better opportunities for learners to discuss differing opinions, the present study identified greater pressure to conform during these sessions as compared to formal teaching sessions. An example of a formal teaching session is an academic half day when usually a large number of residents, many of which are at the same PGY level, are taught by one senior resident or staff. In contrast, during clinical rounds, in the operating room, and in the hallway, there are several trainees who are at different stages of training. These usually represent teams consisting of a medical student, a junior resident, a senior resident, a fellow and a staff member. This creates an obvious power differential and hierarchy within the informal teaching session. In fact, residents reported that they felt little pressure to conform to members of a lower status group compared to any other groups, and greater pressure to conform to their preceptors. About a quarter of the residents in our study said that conformity is likely to occur when making decisions. When asked to give a recommendation for diagnosis and treatment, which are critical decisions, residents may follow others’ suggestions in an effort to mask any lack of knowledge or confusion. Given the many advantages of informal teaching, such as relevancy and flexibility of the topic, it is important that educators facilitate such sessions with the recognition that trainees may feel reluctant to question or challenge information. Educators can openly invite discussion by requesting dissenting opinions and provide encouragement for asking questions. These actions should especially be directed at the more junior members of the group.

Surprisingly, residents in the present study rated that conformity in residency is “rarely” or “sometimes” severe, and in their opinion, resulted in few medical errors. In fact, most residents rated the impact on knowledge and skills as both positive and negative, presumably positive because it meant that they followed correct information and proper skills shared by others. In consideration that others may also lack knowledge or make mistakes, it is negative because residents should check the accuracy of such information and skills demonstration. Another possible explanation is that residents simply chose the option as an expression of uncertainty about their perception of positivity or negativity of the impact on their skills and knowledge acquisition. Or perhaps they feel confident in managing conformity; however, observational studies are necessary to determine how well they are managing incorrect information.

In regards to the implications of our findings, we propose that alterations to the teaching environment have the potential to decrease perceptions of pressure to conform. Since no resident factors were found to be significantly associated with differences in conformity pressure, it may not be advantageous to focus strategies on individuals that would be at increased risk to perceive such pressure. Rather, these strategies may be implemented for trainees at all levels in all programs. Particular focus should be on informal education situations. Of course, our suggestion is not to avoid teaching under informal circumstances. The advantage of topic flexibility and high immediate relevance are something that formal teaching sessions cannot offer. Rather, our suggestion is to “formalize” these sessions. For example, instead of proceeding with the full ad hoc teaching session at the bedside, teachers could outline the most important topics to be discussed in a semi-formal session at a later time. In this way, trainees may achieve at least a minimal level of understanding or competency while preparing for the discussion and some feeling of comfort with the topic. In addition, resident learners could formulate questions to clarify details that were misunderstood. As a result, residents themselves may be able to discuss and answer each other’s questions. With this simple modification, the educator could include off-service residents, change proportions of training levels and hierarchy of participants, avoid presence of patients and their families, establish an expectation of discussion, while still preserving the immediate relevance of the topic, and increase the complexity of the content for discussion. It is not anticipated that this strategy would require additional time, since time spent teaching is only moved, not necessarily expanded. However, as suggested by this research, it is important to note that alteration of the setting (i.e., learning environment) requires an educator’s awareness of the factors relevant to perceived pressure to conform the educator’s willingness to encourage trainee presence and participation during the scheduled session. It is essential that educators take even just the first step in becoming aware that residents may be feeling this pressure.

### Limitations and future directions

Several limitations need to be considered. With the low residency program response rate it is possible that there is a sampling bias that affected the results of the study. Also, we are not able to estimate why respondents reported so few instances of the pressure to conform, or if this in any way might have an impact on the validity of the findings in the previous section of the questionnaire. It is possible, that some of the questions might have been perceived as threatening by residents or that residents perceived reporting these instances as undesirable. In addition, self-report methods tend to yield subjective data; however, since perceptions of pressure are an internal experience, it seemed to be an appropriate method of measurement. We did not ask participants why they would be hesitant to report pressures to conform. Although difficult to do, it would also be desirable to measure rates of conformity behavior in clinical settings. This approach would allow us to quantitatively assess for the presence of underreporting related to self-report of behaviors and attitudes.

In addition to the low response rate, data collection at a single institution further limits generalizability. To make suggestions for other programs within the Canadian postgraduate medical training system and others abroad, multiple similar studies replicating our research are needed.

It is also noted that pressure to conform may not necessarily lead to a medical error or poor medical practice. In fact, conforming to a preceptor’s or peer’s behaviour is relevant to learning from positive role models to gain critical medical knowledge and skills [11]. Future research should investigate residents’ learning and retention upon hearing inaccurate information in situations they feel pressure to conform compared to those where they do not feel this pressure. This understanding would allow us to determine the extent to which conformity may influence residents’ knowledge. Further study of residents’ thoughts and attitudes about conformity through a qualitative approach could reveal the complexity of this phenomenon. At the very least, the present study calls for steps to be taken to mitigate pressure to conform and encourage active engagement in asking questions and exploring knowledge to provide effective teaching for all learners. In summary, the continual pursuit of optimal patient care and reduction of medical errors demands that residents’ pressure to conform be addressed.

## Conclusion

This study confirmed that residents experience pressure to conform in a variety of settings. Factors related to conformity include situational but not personal characteristics. Further research is needed to study the feasibility and effect of mitigation strategies on conformity within standardized scenarios.

## Additional file

Additional file 1: Survey for Residents. Survey. (DOCX 19 kb)

## Abbreviations

CIMGs: - Canadian International Medical Graduates (Canadians studying abroad)
IMGs: - International Medical Graduates
PGME: - Postgraduate Medical Education
PGY: - Postgraduate Year of training
