# Supplementary material for: Experiences of pressure to conform in postgraduate medical education
Source: BMC Med Educ. 2018 Jan 3;18:4. doi: 10.1186/s12909-017-1108-8 (PMC5751422; doi:10.1186/s12909-017-1108-8)
Supplement: Additional file 1: — Survey for Residents. Survey. (DOCX 19 kb) [file 12909_2017_1108_MOESM1_ESM.docx]

Survey for Residents

Year of birth: _____________________ Sex (**Circle one**): Male Female

Residency program: _______________________Year in program (**Circle one**): 1 2 3 4 5 6 7 8

Are you an International Medical Graduate (IMG)? (**Circle one**): Yes No

Are you a Canadian International Medical Graduate (**Circle one**): Yes No

Please, circle the answer that describes your attitudes most:

**1. I avoid settings where people don’t share my values.**

Strongly disagree Disagree Neither agree nor disagree Agree Strongly agree

**2. I can enjoy being with people whose values are different from mine.**

Strongly disagree Disagree Neither agree nor disagree Agree Strongly agree

**3. I would like to live in a foreign country for a while.**

Strongly disagree Disagree Neither agree nor disagree Agree Strongly agree

**4. I like to surround myself with things that are familiar to me.**

Strongly disagree Disagree Neither agree nor disagree Agree Strongly agree

**5. The sooner we all acquire similar values and ideas the better.**

Strongly disagree Disagree Neither agree nor disagree Agree Strongly agree

**6. I can be comfortable with almost all kinds of people.**

Strongly disagree Disagree Neither agree nor disagree Agree Strongly agree

**7. If given a choice, I will usually visit a foreign country rather than vacation at home.**

Strongly disagree Disagree Neither agree nor disagree Agree Strongly agree

**8. A good teacher is one who makes you wonder about your way of looking at things.**

Strongly disagree Disagree Neither agree nor disagree Agree Strongly agree

**9. A good job is one where what is to be done and how it is to be done are always clear.**

Strongly disagree Disagree Neither agree nor disagree Agree Strongly agree

**10. A person who leads an even, regular life in which few surprises or unexpected happenings arise really has a lot to be grateful for.**

Strongly disagree Disagree Neither agree nor disagree Agree Strongly agree

**11. What we are used to is always preferable to what is unfamiliar.**

Strongly disagree Disagree Neither agree nor disagree Agree Strongly agree

**12. I like parties where I know most of the people more than ones where all or most of the people are complete strangers.**

Strongly disagree Disagree Neither agree nor disagree Agree Strongly agree

Residents may find themselves in clinical situations where they feel pressure to go along with other people’s ideas, even when these ideas seem wrong (called conformity). Please indicate the percentage of decisions made during an average rotation in residency when you’ve been in this type of situation _________%.

Please, describe your experience in these settings during both the on-service and off-service rotations:

To what extent have you had this experience when in (**circle one**):

1. **Clinical rounds**

On-service............not applicable almost never rarely sometimes often almost always

Off-service...........not applicable almost never rarely sometimes often almost always

1. **Academic half days**

On-service............not applicable almost never rarely sometimes often almost always

Off-service...........not applicable almost never rarely sometimes often almost always

1. **Discussion with peers**

On-service............not applicable almost never rarely sometimes often almost always

Off-service...........not applicable almost never rarely sometimes often almost always

1. **Operating room**

On-service............not applicable almost never rarely sometimes often almost always

Off-service...........not applicable almost never rarely sometimes often almost always

**17. Other situations (please specify): …………………………………………………………………………………………………………..**

On-service............not applicable almost never rarely sometimes often almost always

Off-service...........not applicable almost never rarely sometimes often almost always

To what extent have you had this experience when with (**circle one**):

**18. Preceptors**

On-service............not applicable almost never rarely sometimes often almost always

Off-service...........not applicable almost never rarely sometimes often almost always

1. **Same year residents**

On-service............not applicable almost never rarely sometimes often almost always

Off-service...........not applicable almost never rarely sometimes often almost always

1. **Residents who are also friends**

On-service............not applicable almost never rarely sometimes often almost always

Off-service...........not applicable almost never rarely sometimes often almost always

1. **Lower year residents**

On-service............not applicable almost never rarely sometimes often almost always

Off-service...........not applicable almost never rarely sometimes often almost always

1. **Higher year residents**

On-service............not applicable almost never rarely sometimes often almost always

Off-service...........not applicable almost never rarely sometimes often almost always

1. **Clerks**

On-service............not applicable almost never rarely sometimes often almost always

Off-service...........not applicable almost never rarely sometimes often almost always

1. **Nurses**

On-service............not applicable almost never rarely sometimes often almost always

Off-service...........not applicable almost never rarely sometimes often almost always

1. **Other health professionals**

On-service............not applicable almost never rarely sometimes often almost always

Off-service...........not applicable almost never rarely sometimes often almost always

1. **Patients**

On-service............not applicable almost never rarely sometimes often almost always

Off-service...........not applicable almost never rarely sometimes often almost always

1. **Family members of patients**

On-service............not applicable almost never rarely sometimes often almost always

Off-service...........not applicable almost never rarely sometimes often almost always

1. **Mixed groups of people**

On-service............not applicable almost never rarely sometimes often almost always

Off-service...........not applicable almost never rarely sometimes often almost always

(Specify groups) __________________________________________________________________________________

**29.** Please describe an event you may have had as a resident when you experienced pressure to go along with others (What did you do, how many people were there, what role did they have

- clerk, junior, senior resident, fellow, staff, nurse): __________________________________________________________________________________

__________________________________________________________________________________

__________________________________________________________________________________

__________________________________________________________________________________

**30.** What did you do? __________________________________________________________________________________

**31.** What was the outcome? __________________________________________________________________________________

**32.** Was there any impact on a patient? __________________________________________________________________________________

**33.** What percentage of residents in your group do you think had a similar experience? ________%

**34.** What percentage of these residents do you think would go along with the pressure? _______%

**35.** If you experienced pressure to go along with others, why did you feel pressured (**circle all that apply**): Short of time Lack of expertise Authority figure present (preceptor)

Other reason __________________________________________________________________________________

Other people present were friends patient family members of patients

**36.** If you have seen another resident experience pressure to go along with others, why do you think they felt pressured (**circle all that apply**):

Short of time Lack of expertise Authority figure present (preceptor)

Other reason __________________________________________________________________________________

Other clinicians present were friends persistent patient persistent family members of patients

**37.** Please rate the severity of this type of issue in residency (**circle one**):

Not at all A bit Somewhat Severe Very

severe severe severe severe

**38.** Has it resulted in any medical errors or adverse events (**circle one**)?

None Few (1-2) Several Many A lot

Please specify ______________________________________________________________________

**39.** In your opinion, what has been the impact of conformity overall on acquiring knowledge in your residency?

Strongly Somewhat Both negative Somewhat Strongly

negative negative and positive positive positive

**40.** In your opinion, what was the impact of conformity overall on acquiring clinical skills in your residency?

Strongly Somewhat Both negative Somewhat Strongly

negative negative and positive positive positive
